# Association of long COVID with health-related Quality of Life and Social Participation in Germany: Finding from an online-based cross-sectional survey

**DOI:** 10.1016/j.heliyon.2024.e26130

**Published:** 2024-02-09

**Authors:** Dominik Schröder, Stephanie Heinemann, Gloria Heesen, Eva Hummers, Tim Schmachtenberg, Alexandra Dopfer-Jablonka, Kai Vahldiek, Frank Klawonn, Sandra Klawitter, Sandra Steffens, Marie Mikuteit, Jacqueline Niewolik, Frank Müller

**Affiliations:** a Department of General Practice, University Medical Center, Göttingen, Germany; b Department of Geriatrics, University Medical Center, Göttingen, Germany; c Department of Rheumatology and Immunology, Hannover Medical School, Germany; d German Center for Infection Research (DZIF), partner site Hannover-Braunschweig, Germany; e Department of Computer Science, Ostfalia University of Applied Sciences, Wolfenbuettel, Germany; f Biostatistics Group, Helmholtz Centre for Infection Research, Braunschweig, Germany; g Deans' Office, Curricular Development, Hannover Medical School, Germany; h Department of Family Medicine, College of Human Medicine, Michigan State University, Grand Rapids, Michigan, USA

**Keywords:** Post-acute COVID-19 syndrome, COVID-19, Quality of life, Patient reported outcome measures, Social participation

## Abstract

**Purpose:**

This study aims to examine the health-related Quality of Life (hrQoL) and social participation in participants with Long COVID compared to participants without symptoms after COVID-19 and participants with no prior SARS-CoV-2 infection.

**Methods:**

A cross-sectional online survey was conducted in Germany. The non-random sample consists of participants 18 years or older. Participants were divided in three groups: Lg COVID with a prior SARS-CoV-2 infection and new or persistent symptoms 28 days after infection, ExCOVID with a prior SARS-CoV-2 infection and without new or persistent symptoms after 28 days, and NoCOVID when participants had no prior SARS-CoV-2 infection. EQ-5D-3L was used as hrQoL measure and the Index for the Assessment of Health Impairments (IMET) to reflect social participation. Descriptive and inferential statistics were performed.

**Results:**

A total of 3188 participants were included in the analysis (1421 Lg COVID, 260 ExCOVID, 1507 NoCOVID). Lg COVID was associated with the lowest EQ-5D-3L index values (p < 0.001), Visual Analogue Scale (VAS) scores (p < 0.001), and IMET (p < 0.001) scores followed by NoCOVID and ExCOVID. After adjusting for sociodemographic and medical conditions in a multivariable model Long COVID was still associated with lower hrQoL compared to NoCOVID (p < 0.001). About 10% of Lg COVID participants showed no health impairments in all EQ-5D dimensions while 51.1% of NoCOVID and 60% of ExCOVID participants showed no health impairments.

**Conclusion:**

This study highlights the impairments of persons with Long COVID on hrQoL and social participation compared to individuals without Long COVID in Germany.

**Trial registration:**

German Clinical Trial Registry, DRKS00026007.

## 1 Background

Worldwide, over 527 million people have been infected, and 6.2 million people have died from severe acute respiratory syndrome coronavirus type 2 (SARS-CoV-2) [1]. Currently, approximately 10%–35% of people who are infected with SARS-CoV-2 have lasting symptoms after a prior infection [2]. Hospitalized COVID-19 patients show a prevalence of about 50% for persisting and/or additional COVID-19 symptoms [[3], [4], [5]]. This post-infectious phenomenon differs among various definitions but can be broadly referred to as “post-acute COVID-19” (symptoms extending three weeks after prior infection) or “Long COVID” (symptoms extending beyond 12 weeks after prior infection) and may affect various organ systems [[6], [7], [8]]. While the exact disease etiology remains unclear, one possible explanation is that immunologic processes are causing persistent inflammation [9]. Those affected describe a variety of different symptoms, such as impaired concentration, fatigue, muscle pain, dizziness, palpitations, mental health problems, or shortness of breath [[10], [11], [12]]. These symptoms can be derived and continue from an acute SARS-CoV-2 infection or may occur after acute COVID-19 disease and may fluctuate in intensity. Treatments targeting the cause of the condition are not yet available [13]. Preventive vaccination against SARS-CoV-2 can likely reduce the risk for Long COVID disease [14].

Studies show that people with Long COVID are usually middle-aged [15]. Thus, the disease strikes them at a time when they are in the workforce and may have families and therefore various social responsibilities. Many studies suggest that Long COVID is associated with a reduction in health-related quality of life (hrQoL) and reduced functionality [[16], [17], [18], [19]]. Social participation as a patient-centered outcome has been widely used in rehabilitation science, studies with mentally or physically impaired persons and geriatric patients but is not limited to these areas [[20], [21], [22]]. Social participation, broadly defined as an individual's engagement in activities that facilitate interaction with others in society or the community, encompasses a wide array of scenarios, extending even to pandemic situations and the profound impact of restrictive measures on daily life during such crises [[23], [24], [25]]. Historically, the discourse on social participation has predominantly centered on individuals with physical, mental, or sensory impairments, particularly in the context of aging populations [[26], [27], [28]]. The medical research community has assimilated the concept of social participation from domains like geriatrics, disability research, and rehabilitation. Within these domains, it is commonly posited that individual illnesses, symptoms, or the aging process itself can modify or constrain an individual's capacity for social participation. Conversely, high social participation is generally associated with favorable health outcomes [[29], [30], [31]]. Enhancing social participation has emerged as a pivotal strategy in addressing the multifaceted challenges posed by an aging society by the WHO [32,33]. In addition to medical and rehabilitative interventions, recognized approaches to augment social participation include optimizing accessibility across diverse services [34]. The effects of these changes may not be fully captured by the construct of hrQoL, but have an impact on both health and daily life [35]. Consequently, social participation goes beyond individually focused approaches such as HRQoL. The reasons for changes in social engagement are diverse and mutually influence the health status of the individual. Many of these influencing factors are adaptive and include aspects such as interpersonal relationships and environmental conditions. Focusing on patient-centered outcomes, such as hrQoL and social participation, provides valuable insights into the social and medical care needs of those affected. Especially in the case of a new and widely spread disease such as Long COVID, characterization of the disease may be useful in terms of therapy design. Research on hrQoL and social participation illustrate a possible bias in the results if other sociodemographic and medical factors are not taken into account [[36], [37], [38]]. These include, for example, age, gender, school education, employment status, migration status, and pre-existing conditions.

Previous studies on hrQoL focus mostly on previously hospitalized SARS-CoV-2 patients [16,39,40]. As hospitalized COVID-19 patients represent only a portion of all long COVID patients, individuals whose previous COVID-19 infection was treated through primary care providers or who even did not see a provider at all were neglected. Besides the lack of knowledge on hrQoL in previously non-hospitalized COVID-19 individuals, there is a need for research on hrQoL among Long COVID in Germany as differences in the health care system including medical treatment guidelines, differences in COVID-19-related restrictions and different cultural background limit the transferability of the results of studies from other countries [8]. In this study, we aim to compare hrQoL and social participation in groups with persons suffering from Long COVID, persons with a SARS-CoV-2 infection but no new or persisting symptoms and persons with no pervious SARS-CoV-2 infection.

The main hypotheses tested in this study are:

(1) Health-related quality of life differs between individuals with and without Long COVID.
(2) Social participation differs between individuals with and without Long COVID.
(3) Various health dimensions are impaired by Long COVID

## 2 Methods

### 2.1 Study design and participants

This study is based on an online survey created by a multi-center interdisciplinary team investigating health and social effects of COVID-19 using a cross-sectional study design with a non-random sample. The digital questionnaire was accessible through the project website (defeat-corona.de) starting in September 2021. A QR code was created and displayed with the link to the project website on posters and flyers in public locations (public libraries, town halls, churches, universities, sports clubs etc.), medical and research centers mostly in Lower Saxony, Germany. Additionally, we shipped information material to randomly selected 400 primary care, internal medicine, radiology and occupational therapy practices across the region of Lower Saxony, Germany. To provide targeted information on social media (e.g., Facebook), we asked selected patient advocacy groups on Long COVID to share the link to the survey on their channels. Additionally, we included the link to our project website with a description of our research project on a national Long COVID website (longcoviddeutschland.org). Participants were included if they a) were 18 years or older and b) resided currently in Germany.

SoSci Survey online platform (SoSci Survey GmbH, Munich, Germany) was used to create the questionnaire. The questionnaire was available only in German language. Before enrollment, participants were asked to provide consent digitally and needed to confirm that they were at least 18 years old. Data was collected without personally identifiable information. Pseudonyms were assigned to participants so that they could take part in follow-up research. Participants could contact the study team by e-mail, telephone and WhatsApp. Further information can be gathered from the study protocol [41].

The study and its data protection plans were assessed and approved by the ethics boards of Hannover Medical School (9948_BO_K_2021) and University Medical Center Göttingen (29/3/21). The study is registered in the German register for clinical trials (DRKS00026007). The survey opened in September 2021 and is still ongoing. The data in this article was retrieved on May 5, 2022 from the SoSci Survey online platform.

### 2.2 Measures

#### 2.2.1 EQ-5D-3L

The three-level version EQ-5D-3L measures the health-related quality of life and consists of two key components: (1) EQ-5D descriptive system, and (2) a visual analog scale (VAS) [31,32]. The descriptive system (1) comprises five items of health (“mobility”, “self-care”, “usual activities”, “pain/discomfort”, “and anxiety/depression”) measuring key health dimensions on a three-point scale (“no problems” [1], “some problems” [2], and “extreme problems” [3]). Responses for all items can be combined into a 5-digit code describing the health state for each subject and health dimension. The first digit of this 5-digit code indicates the health status of the first dimension (“mobility”), the second digit the second dimension (“self-care”), etc. From this 5-digit number, an EQ-5D index value that reflects the overall hrQoL ranging between 0 (worst health) to 1 (best health) can be calculated using a country-specific value set. The VAS (2) contributes to the hrQoL concept by gathering additional information about participants’ self-rated health. Participants indicate their perceived overall health on an analog scale from 0 (“worst health I can imagine”) to 100 (“best health I can imagine”). For more information about the setup of the EQ-5D-3L, refer to the official EQ-5D-3L user manual [42]. The concept of health-related quality of life and the specific item EQ-5D-3L are used to quantify limitations of diseases, cost-effectiveness analyses, and health monitoring [43,44]. The health-related quality of life shows a correlation with the general quality of life and various disease-specific survey instruments [45,46].

#### 2.2.2 Index for the Assessment of Health Impairments (IMET)

The IMET is based on the International Classification of Functioning, Disability and Health [47]. It was initially developed to collect data from people with different chronic diseases and used especially for rehabilitation research. The questionnaire measures the impairments of their social participation (e.g., every day and recreational activities, close relationship to others, family obligations) by using nine items with a Likert-scale with 11 levels (0–10). It does not reflect for the frequency of social activities and four out of nine items does not measure social participation directly. Higher scores indicate greater self-reported impairments to social participation across all nine items. The sum of these nine items shows the overall social participation with a high internal reliability with a Cronbach's alpha of 0.90. Norm data for the IMET is available for health individuals and various chronic conditions from 2014 [48]. The IMET has been used by various authors to measure social impairment during the COVID-19 pandemic [25,[49], [50], [51], [52]].

#### 2.2.3 Primary exposure variable

Participants were grouped in the following three groups: (1) Lg COVID when they stated to have persistent or new symptoms four weeks after a COVID-19 infection confirmed by an antibody test, PCR or positive rapid antigen test; (2) ExCOVID when they stated they have no persistent or new symptoms four weeks after a COVID-19 infection confirmed by an antibody test, PCR or positive rapid antigen test; (3) NoCOVID when the persons were not known to be infected during or before the questionnaire was filled out. Participants that had a recent SARS-CoV-2 infection (less than 29 days ago) were excluded from the analyses.

#### 2.2.4 Covariates

Participants were categorized in age groups (<30 years; 30–49 years; 50–64 years; ≥65 years). School education was classified as low (no or low secondary school diploma), medium (intermediate secondary school diploma) or high (college preparatory) based on the German secondary school graduation and employment status was categorized into full-time employment, part-time employment and unemployed. An employment and a higher school education is known to be associated with higher hrQoL compared to unemployment and lower school education, respectively. Migration status is shown to be associated with lower health care utilization and hrQoL in previous studies [36,53,54]. Regarding Long COVID, migrants have a range of unique risk factors for worse health outcomes, including restricted access to health and vaccination systems [55]. Migration background was included as a covariate using the definition of the German Federal Employment Agency [56].

Comorbidities are associated with reduced hrQoL and could confound the results. In order to avoid collinearity and to achieve a larger sample size of the comorbidities, the individual comorbidities were categorized into the following groups: Heart diseases (high blood pressure, heart failure, coronary heart disease or cardiac arrhythmia), autoimmune diseases (ulcerative colitis, chronic hepatitis, psoriasis, various allergies, neurodermatitis, thyroid diseases, rheumatoid arthritis or other rheumatic diseases). If a person stated that they have or had at least one cancer disease, they were categorized into the variable “cancer”.

Participants were considered vaccinated if they received at least one vaccine shot against COVID-19 regardless of the available vaccines. In persons with a COVID-19 infection, the vaccine status was subdivided if they were vaccinated before or after the onset of first COVID-19 symptoms.

### 2.3 Data analysis

For statistical analysis, participants were excluded if they did not state their pseudonym at the beginning of the survey. The entry of the pseudonym was required to proceed with the questionnaire. If a pseudonym was duplicated, the questionnaire with the least missing values based on all answers was used. Participants with missing data in EQ-5D-3L index values, EQ-5D VAS and IMET scores were excluded from statistical analysis.

Sociodemographic factors were presented with number of observations in each category for categorized data or mean and standard deviation for numeric data (Table 1). Unadjusted EQ-5D index values, EQ-5D VAS scores and IMET scores with its subscales were compared using the Kruskal-Wallis test and reported with mean and standard deviation (Table 2). Post-hoc analysis was performed using the Mann-Whitney-U-test (Bonferroni adjusted). The association between hrQoL and social participation was additionally operationalized using the Spearman's rank correlation coefficient with EQ-5D index values, EQ-5D VAS scores and IMET scores (with its subscales) (Table S1). To gain additional insight which health domains are affected in persons with Long COVID the impairments levels for each of the five health dimensions were reported and tested for difference between the groups using the Fisher-Freeman-Halton Exact test (Table 3). To determine which of the groups differ from each other we used Fisher's exact test. Additionally, the ten most frequent health profiles were descriptively reported with mean of the EQ-5D VAS and IMET score to show the variability of health impairment combinations and describe the effect the health impairments combination on hrQoL and social participation (Tables S2 and S3). The association of Long COVID on subjective hrQoL (EQ-5D VAS scores) adjusted for social demographic and medical factors was quantified using a general linear model with a poisson distribution (Table 4). As hrQoL data is considerably skewed, a poisson distribution is typically used [[57], [58], [59], [60]]. Decrement values were used (decrement value = 100 – EQ-5D VAS scores) as dependent variable. A negative regression coefficient represents a higher EQ-5D VAS score compared to the reference category and positive regression coefficient represents a lower EQ-5D VAS score compared to the reference category. Multicollinearity was evaluated using the variance inflation factor where values over four can indicate excessive multicollinearity [61]. Regression coefficients with corresponding 95% confidence intervals were reported. Two models were tested. Model one contained the variable COVID type adjusted for sociodemographic (age, sex, school education, employment status and migration status) and medical (heart disease, autoimmune disease, depression and cancer) variables. The second model additionally contained IMET scores. The added explained value between Model one and Model two was tested using drop-in-deviance test.

**Table 1:** Characteristics of participants.

	All n = 3188 (n%)	Lg COVID n = 1421 n (%)	ExCOVID n = 260 n (%)	NoCOVID n = 1507 n (%)
Age (years) (mean (SD))	42.2 (12.7)	42.8 (12.2)	38.8 (12.9)	42.2 (13.0)
18-29	623 (19.5)	247 (17.4)	76 (29.2)	300 (19.9)
30-49	1542 (48.4)	699 (49.2)	121 (46.5)	722 (47.9)
50-64	919 (28.8)	441 (31.0)	53 (20.4)	425 (28.2)
65+	100 (3.1)	33 (2.3)	9 (3.5)	58 (3.8)
Not answered	4 (0.1)	1 (0.0)	1 (0.3)	2 (0.1)
Sex				
Male	870 (27.3)	299 (21.0)	99 (38.1)	472 (31.3)
Female	2302 (72.2)	1113 (78.3)	160 (61.5)	1029 (68.3)
Not answered	16 (0.4)	9 (0.6)	1 (0.4)	6 (0.4)
School education^b^				
Low	137 (4.3)	84 (5.9)	9 (3.5)	44 (2.9)
Middle	821 (25.8)	488 (34.3)	57 (21.9)	276 (18.3)
High	2212 (69.4)	839 (59.0)	193 (74.2)	1180 (78.3)
Not answered	18 (0.6)	10 (0.7)	1 (0.3)	7 (0.5)
Employment status				
Full-time	1718 (53.9)	738 (51.9)	145 (55.8)	833 (55.3)
Part-time	919 (28.8)	433 (30.5)	75 (28.8)	411 (27.3)
Unemployed	530 (16.6)	235 (16.5)	40 (15.4)	255 (16.9)
Not answered	21 (0.7)	15 (1.1)	0 (0.0)	6 (0.4)
Migration background^c^	225 (7.1)	98 (6.9)	17 (6.5)	110 (7.3)
Comorbidities^a^				
Cancer	1061 (33.3)	448 (31.5)	96 (36.9)	517 (34.3)
Heart disease	514 (16.1)	253 (17.8)	23 (8.8)	238 (15.8)
Autoimmun	1149 (36.0)	559 (39.3)	78 (30.0)	512 (34.0)
Depression	279 (8.8)	134 (9.4)	17 (6.5)	128 (8.5)
Vaccination	2589 (81.2)	1093 (90.6)	186 (81.6)	1310 (90.9)
Before first Symptoms	–	271 (19.1)	88 (33.8)	–
Time from beginning of the infection to survey in weeks (mean (SD))	–	53.2 (18.8)	29.6 (26.3)	–

a multiple selection possible.

b based on German secondary school education.

c according to the definition of the German Federal Employment Agency.

**Table 2:** EQ-5D-3L index values, EQ-5D VAS and IMET scores by COVID categorization.

	Lg COVID	ExCOVID	NoCOVID	p value^1^
EQ-5D-3L index values	0.66 (0.23)	0.90 (0.14)	0.86 (0.20)	<0.001
EQ-5D VAS scores	57.6 (22.2)	85.8 (13.0)	78.7 (19.9)	<0.001
Social participation (IMET)^2^	33.5 (21.6)	11.9 (13.2)	19.7 (19.4)	<0.001

Usual activities of daily life	1.5 (2.2)	0.2 (1.0)	0.7 (1.8)	<0.001
Family and domestic responsibilities	3.1 (2.9)	0.8 (1.7)	1.5 (2.5)	<0.001
Getting things done outside of home	3.2 (2.9)	0.8 (1.7)	1.6 (2.5)	<0.001
Daily tasks and obligations	4.4 (3.3)	0.9 (1.7)	1.8 (2.7)	<0.001
Recreation and leisure	4.7 (3.0)	2.0 (2.7)	3.1 (3.1)	<0.001
Social activities	4.2 (3.2)	2.0 (2.9)	3.4 (3.3)	<0.001
Close personal relationships	2.9 (2.9)	1.3 (2.2)	2.2 (2.7)	<0.001
Sex life	3.7 (3.5)	1.5 (2.5)	2.5 (3.2)	<0.001
Stress and extraordinary strain	5.3 (3.1)	2.5 (2.4)	3.3 (2.8)	<0.001

Post-hoc analysis revealed significant differences between all groups in all included scales and subscales; Data is mean (SD); ^1^Kruskal-Wallis test (Bonferroni adjusted when reported in IMET subscales);^2^higher values indicate less social participation.

**Table 3:** EQ-5D impairment domains.

All (n = 2875)	Lg COVID (n = 1421)	ExCOVID (n = 260)	NoCOVID (n = 1507)	p value^1^
Mobility impairments^a,b^	480 (33.8)	8 (3.1)	168 (11.1)	<0.001
Self-care impairments^a,b^	105 (7.4)	0 (0)	62 (4.1)	<0.001
Usual activities^a,b^	987 (69.5)	24 (9.2)	263 (17.5)	<0.001
Pain/discomfort^a,b^	1149 (80.9)	53 (20.4)	534 (35.4)	<0.001
Anxiety/depression^a^	856 (60.2)	69 (26.5)	450 (29.9)	<0.001

Data is n (%) of participants with at least one impairment; ^1^Fisher-Freeman-Halton Exact Test; ^a^: significant Fisher's exact test between Lg COVID/ExCOVID and LgCOVID/NoCOVID; ^b^: significant Fisher's exact test between ExCOVID/NoCOVID.

**Table 4:** Poisson regression coefficients with corresponding 95%-CIs predicting EQ-5D VAS disutility scores.

	Model 1^a^		Model 2^a^	
	Coefficient (95%-CI)	p-value	Coefficient (95%-CI)	p-value
COVID type				
NoCOVID (ref)	–		–	
Lg COVID	0.68 (0.67; 0.69)	<0.001	0.43 (0.41; 0.44)	<0.001
ExCOVID	−0.37 (−0.41; −0.34)	<0.001	−0.20 (−0.23; −0.16)	<0.001
Gender				
Male	–		–	
Female	−0.03 (−0.05; −0.01)	<0.001	0.02 (0.01; 0.04)	0.002
Age (per year)	0.00 (0.00; 0.00)	<0.001	0.00 (−0.00; 0.00)	0.37
School education				
Low (ref)	–			
Middle	−0.10 (−0.13; −0.07)	<0.001	−0.06 (−0.09; −0.03)	<0.001
High	−0.15 (−0.18; −0.12)	<0.001	−0.07 (−0.10; −0.04)	<0.001
Employment status				
Full-time (ref)	–		–	
Part-time	0.08 (0.07; 0.10)	<0.001	0.03 (0.02; 0.04)	<0.001
unemployed	0.28 (0.27; 0.30)	<0.001	0.10 (0.09; 0.13)	<0.001
Migration status				
No migration background (ref)	–		–	
Migration background	−0.09 (−0.11; −0.06)	<0.001	−0.09 (−0.12; −0.07)	<0.001
Disease (no = ref)				
Heart disease	0.05 (0.03; 0.06)	<0.001	0.06 (0.04; 0.08)	<0.001
Depression	0.27 (0.25; 0.29)	<0.001	0.10 (0.08; 0.12)	<0.001
Cancer	−0.04 (−0.06; −0.03)	<0.001	−0.01 (−0.03; 0.00)	0.08
Autoimmune disease	0.11 (0.10; 0.12)		0.07 (0.06; 0.08)	<0.001
IMET (per Score)	–		0.02 (0.02; 0.02)	<0.001

ref: reference category; CI: confidence interval; IMET: Index for the Assessment of Health Impairments.

a positive regression coefficient indicates a lower hrQoL.

All statistical analysis and graphical illustrations were performed using the software R (Version 4.1.2). We used the package ggplot2 (Version 3.3.6) to create visual illustrations of the results [62]. EQ-5D-3L index values were calculated with the R package *eq5d* (Version 0.11.0) [63] using the most recent German dataset derived from VAS data. Results were considered statistically significant with a p-value <0.05. When testing the subscales of the IMET, the p values were adjusted using the Bonferroni method.

## 3 Results

**Fig. 1:**
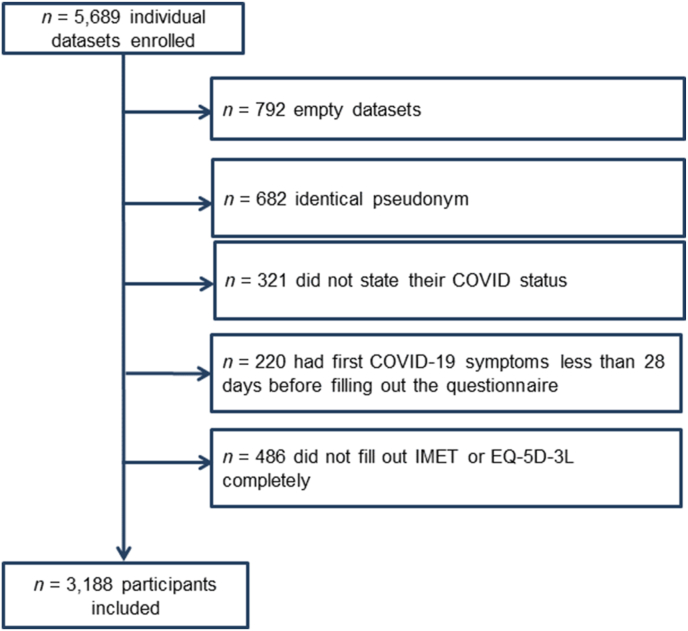
Flowchart of included participants.

In total 5689 participants were enrolled and after applying inclusion and exclusion criteria 3188 participants (56.1%) were included in the final analysis (Fig. 1). The first participant was recruited on the 19th of September 2021 and the last participants on 4^th^ May 2022. Most included participants reported no prior SARS-CoV-2 infection (n = 1507), followed by participants with Long COVID (n = 1421) and ExCOVID (n = 260).

### 3.1 Participant characteristics

Participants were mostly female (n = 2302 (72.2%)) and between 30 and 49 years old (n = 1542 (48.4%)). ExCOVID participants were younger and had a higher proportion of male participants compared to Lg COVID and NoCOVID participants (Table 1). About 7% of all participants (n = 225) had a migration background. As comorbidities, cancer followed by autoimmune disease, heart disease and depression were most prevalent. Autoimmune diseases were significantly more prevalent in participants with Long COVID. About nine of ten participants with Lg COVID and No COVID had at least one vaccine shot against COVID-19 while only about 8 out of 10 ExCOVID participants were vaccinated. When investigating the vaccination of participants with a past COVID infection further, 19.1% (n = 271) of Lg COVID participants and 31.1% (n = 88) of ExCOVID participants were vaccinated before first COVID-19 symptoms occurred. Out of all included participants, 37.3% (n = 1188) stated that their work, residency or medical care is located in Lower Saxony.

### 3.2 Health-related quality of life and social participation

The unadjusted mean EQ-5D-3L index values scored higher than the unadjusted corresponding mean EQ-5D VAS scores in all groups. A significant difference in unadjusted EQ-5D-3L index values, EQ-5D VAS scores, IMET scores and its subscales was found between the three groups. Pairwise post-hoc analysis revealed significant differences between all groups in all included scales and subscales. Across all included measures ExCOVID participants were associated with the best health outcomes, followed by NoCOVID participants. Lg COVID participants had the worst health outcomes (Table 2).

Between unadjusted EQ-5D-3L index values and EQ-5D VAS scores a correlation coefficient of 0.74 was observed. Both hrQoL measures correlated comparably with IMET scores (table SI 1). When stratified after COVID type, Lg COVID participants (Index: −0.72; VAS: −0.70) show the highest correlation between hrQoL and IMET scores followed by NoCOVID (Index: −0.56; VAS: −0.53) and ExCOVID (Index: −0.45; VAS: −0.34). Investigating different domains of the IMET individually, the highest correlation with hrQoL was found in the domain representing “Family and domestic responsibilities” followed by “Daily tasks and obligations”.

### 3.3 Impaired health domains

Between the three groups Lg COVID, ExCOVID and NoCOVID the distribution of impairments levels differed across all impairment domains (Table 3). Lg COVID was associated in all health domains with the highest prevalence of impairments compared to ExCOVID and NoCOVID. The domains “usual activities”, “pain/discomfort” and “anxiety/depression” were prevalent in over 50% of all Long COVID participants (Table 3). Post-hoc analysis revealed a difference between all groups in all dimensions except for “anxiety/depression” between the groups ExCOVID and NoCOVID.

For the ten most frequent health profiles EQ-5D VAS and IMET scores are presented stratified after the COVID Groups in Tables S2 and S3.

### 3.4 Multivariable analysis

**Fig. 2:**
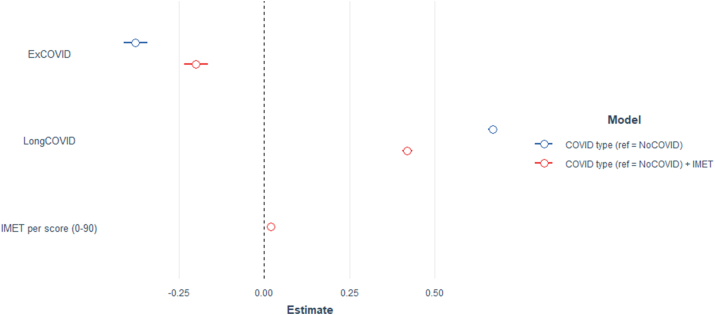
Possion regression coefficients predicting EQ-5D VAS disutility scores comparing two models (reference: NoCOVID) Point estimates with 95% confidence intervals; model adjusted for age (continuous), gender, migration status, school education and comorbidities (heart disease, autoimmune disease, cancer, depression); negative regression coefficients indicate higher health-related Quality of Life.

Multivariable analysis revealed a significant association between a history of COVID and the disease course after adjusting for sociodemographic (age, gender, school education, employment status and migration status) and medical (heart disease, autoimmune disease, depression and cancer) variables. Lg COVID was associated with a lower hrQoL (β 0.67, 95% CI [0.66; 0.68], p < 0.001) and ExCOVID (β −0.38, 95% CI [−0.41; −0.34], p < 0.001) with higher hrQoL compared to the reference group NoCOVID. All sociodemographic and medical variables were significantly associated with hrQoL (Table 4). When IMET scores were included into the model as an independent variable, the model improved significantly (*X*^2^_df = 1_ = 14,833, p < 0.001). With included IMET scores (β 0.02, 95% CI [0.02; 0.02], p < 0.001) reflecting social impairments, Long COVID (β 0.42, 95% CI [0.40; 0.43], p < 0.001) and ExCOVID (β −0.20, 95% CI [−0.23; −0.17], p < 0.001) were still associated significantly with EQ-5D VAS scores but the association shifted towards the no effect line (Fig. 2). In the second model, all sociodemographic and medical variables except for cancer (p = 0.08) were associated significantly with the dependent variable (Table 4). Variance inflation factor indicated non-collinearity in both regression models and ranged between 1.00 and 1.18 between all variables in both regression models.

## 4 Discussion

This cross-sectional study compares hrQoL and social participation among persons with Long COVID symptoms (n = 1421) between persons with no prior SARS-CoV-2 infection (n = 1507) and persons with a prior SARS-CoV-2 infection without new or persisting symptoms (n = 260). Scores for hrQoL and social participation differed across all three groups. Participants with Long COVID exhibited the lowest HRQoL and the highest impairments in social participation compared to participants with NoCOVID and ExCOVID, even after adjusting for sociodemographic factors, comorbidities, and social participation. Health profiles according to EQ-5D-3L were heterogenous in Long COVID participations where Ex- and NoCOVID showed dominantly no health impairments.

Social participation among individuals with Long COVID in our study was significantly more impaired when compared to the NoCOVID and ExCOVID control groups. Additionally, the impairment was significantly higher than among other chronic diseases such as diabetes [64] or inflammatory bowel disease [65]. Similarly, high scores were seen in persons with chronic psychiatric disorders during the early phases of the COVID-19 pandemic [49]. The highest scores were found in the subdomains stress and extraordinary strain. This suggests that the experience of having limited functioning and participation in social life is associated with significant psycho-emotional stress. In addition, the lack of potent treatments and rehabilitative capacities, as well as uncertainties regarding prognosis, may contribute to this stress. Still, the IMET questionnaire only measures social participation indirectly. Instruments that specifically measure participation of younger chronically sick persons, including working life, could generate additional knowledge. Furthermore, qualitative approaches seem preferable in order to gain a better understanding of the experiences that individuals with Long COVID have in accessing care. Results could contribute to better and more adequate understanding of the disease and its burden for the affected, which in turn could aid in the development of treatment options. In addition, support services should be offered to make participation possible despite low health-related quality of life, e.g., by offering occupational therapy to cope with everyday tasks. The reference cohorts (NoCOVID and ExCOVID) had only slightly elevated scores in the areas of recreation and leisure as well as social activities. This seems understandable in light of the COVID-19 pandemic and the high COVID-19 incidence rates at the time of the study. Similar scores on the IMET were also observed in a normal population sample at the onset of the pandemic [49] and in a group at a particularly high risk for a severe COVID-19 disease progression [25].

The hrQoL of our study can be compared with other studies using the EQ-5D in Germany. The German reference population from the years 2014 and 2015 shows a mean VAS scores of 79.45 with 36.4% stating no impairments using the EQ-5D-3L [66]. Our study shows comparable results with about 34.9% of all participants having no impairments. After stratifying for COVID infection and disease course, most of the participants with Long COVID showed impairments (90.8%). In the German older adults population (65 years or older), a mean EQ-5D VAS score of 73.2 was observed in 2018 [67]. Compared to the previously described German older adults population, our Lg COVID cohort shows lower EQ-5D VAS scores (mean 57.6) indicating less hrQoL despite a younger mean age of 42.8 years. A less recent study with representative data for the German population from 2012 to 2014 shows a EQ-5D VAS of 84.3 which is comparable to our ExCOVID group with a EQ-5D VAS score of 85.8 [68]. Since the current EQ-5D reference cohort in Germany was recruited between 2014 and 2015, which is used for the calculation of the EQ-5D index values, the current calculation of the values could be out of date and distort them especially in the pandemic situation. In addition, Lg COVID is associated with a wide variety of symptoms, with the EQ-5D poorly reflecting cognition and communication impairments [69]. In our study, participants with a previous SARS-CoV-2 infection showed fewer health impairments than participants without a previous SARS-CoV-2 infection even after adjusting for various sociodemographic and medical factors. One possible explanation could be that people who have already had COVID-19 are less anxious and no longer restrict their everyday life out of fear of COVID-19. These findings need to be investigated further.

Compared to other studies investigating hrQoL in COVID-19 survivors, Taboada et al. observed a reduction of EQ-5D mean index value from 0.97 to 0.71 and mean EQ-5D VAS score from 87.6 to 66.4 before and 6 months after a severe COVID-19 course [70]. Both the EQ-5D index value and EQ-5D VAS score were even lower in our study compared to the 6-month follow-up in the Taboada et al. study. This could be due to the fact that not all severe COVID-19 infections result in Long COVID. Of those hospitalized for SARS-CoV-2 infection, 30% had persistent symptoms at 6 months after infection and 25.3% had persistent symptoms at 12 months [71]. The incidence of Long COVID is shown to vary depending on various factors including vaccination status for Long COVID [72] and SARS-CoV-2 variants [73] which changed during the pandemic. Our study had a high heterogeneity of affected EQ-5D dimensions in persons with Long COVID, similar to the findings of Tabacof et al. [17]. In participants with Long COVID hundreds of different biomedical finding have been documented across multiple organ systems and overlap with preexisting conditions [74] which would explain the various affected health dimensions.

This study has some limitations that need to be considered when interpreting the results. The study design, a cross-sectional study, does not allow to establish a temporal relationship between influencing factors and outcome. Therefore, no causality can be determined in the studied population. Our sample differs from the overall German population and is therefore not representative. Our cohort consists of more female, younger and higher-educated participants than the German population. The used questionnaire is only available online and in German language, therefore participants facing barriers to access the internet and with limited German proficiency were unlikely to participate. The questionnaire is self-assessed and therefore participants stated themselves if they had a SARS-CoV-2 infection. Participants in the NoCOVID group may had an asymptomatic SARS-CoV-2 infection. According to a meta-analysis by Ma et al. (2021) only 0.25% of individuals with confirmed COVID-19 diagnosis were asymptomatic [75]. The IMET questionnaire does not reflect actual social participation. Instead, the perceived social participation is reflected. The IMET questionnaire has its origin in rehabilitation science and focuses on social and functional abilities, therefore about half of the items does not reflect the social participation. The usage of a different instrument may gather different results depending on the underlying social participation definition and framework. The inclusion of additional covariates into our multivariable regression models like income and family size could influence the effect of Long COVID on hrQoL and social participation. Analysis of health states between the groups impairment domains and health stated were not adjusted for possible confounder. COVID-19 related restrictions across Germany are based on a central scope of action but can differ between federal states in terms of implementation. Therefore, the time point which varies between participants could have impacted our results due to various COVID-19 related restriction (i.e., starting December 1^st,^ 2021 some states in Germany applied a rule that a third vaccination was required to participate in public events such as concerts). For example, Heesen et al. could observe increased social participation after a complete COVID-19 vaccination [25]. Despite the influence of the pandemic situation our results give additional insight to social and health impairments of participants which reflect health care utilization and needs for treatment at the given time-point. A follow-up study could give additional insights on the effects of the pandemic as most measures to minimize the infection risk were repealed by February 1^st,^ 2023 in Germany (i.e., quarantine for persons infected with SARS-CoV-2 and mask obligation in public transportation). In this study, the EQ-5D-3L was employed to quantify HRQoL. Using the EQ-5D-5L would have yielded more precise results [76]. EQ-5D was used as the only hrQoL measure. The usage of additional hrQoL measures like the Quality of Life Scale (QOLS) or Short-Form-36 (SF-36) could gain additional insights and be used in a sensitivity analysis [77,78]. The ratio of the included participants between the COVID groups, especially the ExCOVID group, was uneven and does not reflect the distribution in the population. Due to the convenience sample, the effect could be overestimated, e.g. if recruited participants with Long COVID were predominantly severe cases. On the other hand, persons with low school education and persons with migrant background are underrepresented in our cohort. This could lead to an underestimation of the effect of Long COVID on hrQoL.

While many other studies on Long COVID have recruited initially hospitalized patients with severer conditions, the vast majority in our sample had developed Long COVID after a rather mild initial acute phase. Our sample is thus more representative for the impairments in hrQoL and social participation for the overall Long COVID population. The inclusion of two separate control groups with fully recovered COVID-19 and NoCOVID is another distinctive feature of this study. Further strengths of the study include the large sample size and that both instruments for the outcome measures were not only validated in the German language and are widely used in health research but also have published reference values for normal population.

## 5 Conclusions

Long COVID is associated in our cohort with impaired social participation and lower-levels of hrQoL compared to participants without new or persisting symptoms after a SARS-CoV-2 infection or no prior infection. Over 50% of Long COVID participants stated impairments in the dimensions usual activities, pain/discomfort and anxiety/depression. With a lower hrQoL, as observed in persons with Long COVID, greater limitations in social participation were also noted. The limitations of social participation are manifold and in addition to being a burden on persons with Long COVID, they are also a burden on society. The observed lower health-related quality of life is observed with limitations in a wide variety of dimensions. Further research should further specify Long COVID with its disease mechanisms to explain the heterogeneity between individuals. The heterogeneity of limitations and disease symptoms between sufferers requires adapted therapies for the care of Long COVID sufferers. Due to the complexity of the disease, the limitations and the individual life situations of persons with Long COVID, interdisciplinary collaboration is essential.

## Funding

This project is part of the DEFEAT-Corona Project funded by the 10.13039/501100008530European Regional Development Fund (ZW7-85152953).

## Ethics approval

The study was conducted according to the guidelines of the Declaration of Helsinki and approved by the Institutional Review Board of Hannover Medical School (9948_BO_K_2021) and University Medical Center Göttingen (29/3/21). The study is registered in the German register for clinical trials (DRKS00026007).

## Consent to participate

Informed consent was obtained from all individual participants included in the study.

## Data availability statement

Data will be made available on request.

## CRediT authorship contribution statement

**Dominik Schröder:** Writing – original draft, Visualization, Validation, Methodology, Formal analysis. **Stephanie Heinemann:** Writing – original draft, Supervision, Project administration, Methodology, Conceptualization. **Gloria Heesen:** Writing – review & editing, Data curation. **Eva Hummers:** Writing – review & editing, Funding acquisition, Conceptualization. **Tim Schmachtenberg:** Writing – review & editing. **Alexandra Dopfer-Jablonka:** Writing – review & editing, Funding acquisition, Conceptualization. **Kai Vahldiek:** Writing – review & editing, Formal analysis, Data curation. **Frank Klawonn:** Writing – review & editing, Funding acquisition. **Sandra Klawitter:** Writing – review & editing. **Sandra Steffens:** Writing – review & editing, Funding acquisition. **Marie Mikuteit:** Writing – review & editing, Data curation. **Jacqueline Niewolik:** Writing – review & editing, Data curation. **Frank Müller:** Writing – original draft, Validation, Project administration, Methodology, Funding acquisition, Conceptualization.

## Declaration of competing interest

The authors declare that they have no known competing financial interests or personal relationships that could have appeared to influence the work reported in this paper.
