# Supplementary material for: Association of long COVID with health-related Quality of Life and Social Participation in Germany: Finding from an online-based cross-sectional survey
Source: Heliyon. 2024 Feb 9;10(4):e26130. doi: 10.1016/j.heliyon.2024.e26130 (PMC10877341; doi:10.1016/j.heliyon.2024.e26130)
Supplement: Multimedia component 1 [file mmc1.pdf]

defeatcorona1 → dftc 07.05.2023, 19:53

Seite 01

EI01

Liebe Studienteilnehmer:innen,

mit dem neuen Projekt DEFEAT Corona soll das Long COVID Syndrom weiter erforscht werden.

Das Projekt wird von der Medizinischen Hochschule Hannover, der Universität Göttingen und der Ostfalia Hochschule für angewandte Wissenschaften Wolfenbüttel durchgeführt und wird von der EU (EFRE) gefördert. Wir laden alle Menschen über 16 Jahre zur Teilnahme an der Befragung ein. Wir suchen Sie, wenn Sie:

- keine Corona Infektion (als Vergleichsgruppe),
- eine Corona Infektion oder Spätsymptome nach einer Corona Infektion hatten oder haben.

Vielen Dank für Ihre Teilnahme! Jede Teilnahme hilft uns, das Long COVID Syndrom besser zu verstehen und können die Behandlung und das weitere Outcome von Corona Patient:innen positiv beeinflußen.

Noch Fragen? Wir stehen Ihnen unter info@defeat-corona.de gerne zur Verfügung

EI02

Was ist DEFEAT Corona?

In dem Projekt DEFEAT Corona wird untersucht, wie es Menschen geht, die eine COVID Infektion durchgemacht haben. Ca. 10% der Patient:innen, die Corona hatten, leiden unter Langzeitfolgen. Derzeit gibt es noch wenige Informationen zum sogenannten Long COVID Syndrom.

Wir laden alle Menschen, ab 16 Jahren, zur Teilnahme ein, egal ob Sie eine Corona Erkrankung hatten oder nicht. Im Fragebogen sind Fragen zu gesundheitlichen Beschwerden während, nach oder auch ohne eine Corona Infektion und zu Ihrer sozialen Situation, Vorerkrankungen und Lebensqualität.

Wir würden uns sehr freuen, wenn Sie uns ihre Kontaktdaten hinterlassen würden, damit wir Sie ggf. zu einer weiterführenden Untersuchung (Befragung, Interview, Spezialsprechstunde) einladen können. Dafür erhalten Sie dann gesonderte Informationen. (Teilnehmer:innen Information)

Die Teilnahme an dieser Studie ist freiwillig. Ihre Daten werden in pseudonymisierter Form auf dem Server des Rechenzentrums der MHH gemäß den datenschutzrechtlichen Vorschriften, insbesondere der DSGVO, gespeichert und verarbeitet. Weitere Informationen entnehmen Sie bitte dem Datenschutzkonzept (Datenschutzkonzept).

Eine Beendigung der Studienteilnahme ist jederzeit ohne Angabe von Gründen möglich. Im Falle eines Widerrufs werden Ihre Daten gelöscht, sofern Sie nicht erlauben, dass Ihre Daten in anonymisierter Form für die Studie weiterverwendet werden dürfen.

Haben Sie Fragen zur Studie?

Melden Sie sich gerne unter info@defeat-corona.de oder unter 0160 9779 1528.

EI43

Mit dem Absenden dieses Formulars bestätige ich:

- Ich habe die Teilnehmer:inneninformationen gelesen und verstanden. Wenn ich Fragen habe, wurden sie entweder per E-Mail oder telefonisch beantwortet.
- Ich nehme freiwillig teil. Ich kann jederzeit und ohne Angaben von Gründen meine Einwilligung widerrufen, ohne dass mir daraus irgendwelche Nachteile entstehen.
- Meine Daten werden nur für wissenschaftliche Zwecke von der MHH, der UMG und der Ostfalia Hochschule ausgewertet.
- Meine Kontaktdaten werden gesondert gespeichert und nicht mit meinen Angaben in den Fragebögen vermischt (pseudonymisierte Datenverwaltung). Meine Kontaktdaten werden nicht weitergegeben, außer ich stimme nochmal gesondert dafür zu.

◯ Ja, ich möchte teilnehmen und bin 18 oder über 18 Jahre alt.

◯ Ja, ich möchte teilnehmen und bin 16 oder 17 Jahre alt.

◯ Nein, ich möchte nicht teilnehmen.

2 aktive(r) Filter

Filter EI43/F1
Wenn eine der folgenden Antwortoption(en) ausgewählt wurde: **2**
Dann nach dem Klick auf "Weiter" den Text **EI13** anzeigen und das Interview beenden

Filter EI43/F2
Wenn eine der folgenden Antwortoption(en) ausgewählt wurde: **3**
Dann Frage/Text **EI44** später im Fragebogen anzeigen (sonst ausblenden)

Seite 03

jump1

EI44

Redundantbitte raus

Bitte lassen Sie Ihre:n Erziehungsberechtigte:n hier zustimmen.

Ich bin Erziehungsberechtigte:r der teilnehmenden Person und mit der Teilnahme meiner Tochter/ meines Sohnes an der Studie einverstanden (vor der endgültigen Teilnahme bei ErgoLoCo findet auch nochmals eine gesonderte Aufklärung statt).

- ◯ Ja
- ◯ Nein

1 aktive(r) Filter

Filter EI44/F1
Wenn eine der folgenden Antwortoption(en) ausgewählt wurde: **2**
Dann nach dem Klick auf "Weiter" den Text **EI13** anzeigen und das Interview beenden

Seite 04

patcode r

PHP-Code

```
$ref = strtoupper(reference()); //reference-Wert in Großbuchstaben umwandeln
if (reference() != '') {goToPage ('kontakt');}
put('patcode', $ref);
```

EI28
redundant bitte raus

Erstellung eines Pseudonyms

Wir möchten Sie bitten, ein Pseudonym für diese Studie zu erstellen. Das Pseudonym besteht aus Buchstaben und Zahlen.

Die Daten in der Studie werden nur mit dem Pseudonym verarbeitet, Sie sind damit nicht identifizierbar. Falls Sie weitere Fragebögen beantworten, können wir die Antworten miteinander verbinden, ohne persönliche Daten von Ihnen zu erheben. Bitte merken Sie sich Ihr Pseudonym, um es ggf. später wieder anzugeben.

Mehr über die Erstellung des Pseudonyms erfahren Sie hier.

EI08
redundant bitte raus

Geburtsmonat der Mutter [Bitte auswählen]

EI09
redundant bitte raus

Erster Buchstabe Vorname der Mutter [Bitte auswählen]

EI10
redundant bitte raus

Geburtsmonat Vater [Bitte auswählen]

EI11
redundant bitte raus

1. Buchstabe Vorname Vater [Bitte auswählen]

EI12

Letzte Ziffer des eigenen Geburtsjahres [Bitte auswählen]

Seite 06

PHP-Code

```
// PS1 prüfen und ggf. umcodieren;
if (value('EI08')=='-9') {$Kode1 = "--";
} else {
for ($i = 1; $i <= 9; $i++) {
    if (value('EI08')==$i) {$Kode1 = "0".$i;
}}}
if (value('EI08')=='10') {$Kode1 = "10";}
if (value('EI08')=='11') {$Kode1 = "11";}
if (value('EI08')=='12') {$Kode1 = "12";}

// PS2 prüfen und ggf. umcodieren;
if (value('EI09')=='-9') {$Kode2 = "-";
} else {
if (value('EI09')==1) {$Kode2 = "A";}
if (value('EI09')==2) {$Kode2 = "B";}
if (value('EI09')==3) {$Kode2 = "C";}
if (value('EI09')==4) {$Kode2 = "D";}
if (value('EI09')==5) {$Kode2 = "E";}
if (value('EI09')==6) {$Kode2 = "F";}
if (value('EI09')==7) {$Kode2 = "G";}
if (value('EI09')==8) {$Kode2 = "H";}
if (value('EI09')==9) {$Kode2 = "I";}
if (value('EI09')==10) {$Kode2 = "J";}
if (value('EI09')==11) {$Kode2 = "K";}
if (value('EI09')==12) {$Kode2 = "L";}
if (value('EI09')==13) {$Kode2 = "M";}
if (value('EI09')==14) {$Kode2 = "N";}
if (value('EI09')==15) {$Kode2 = "O";}
if (value('EI09')==16) {$Kode2 = "P";}
if (value('EI09')==17) {$Kode2 = "Q";}
if (value('EI09')==18) {$Kode2 = "R";}
if (value('EI09')==19) {$Kode2 = "S";}
if (value('EI09')==20) {$Kode2 = "T";}
if (value('EI09')==21) {$Kode2 = "U";}
if (value('EI09')==22) {$Kode2 = "V";}
if (value('EI09')==23) {$Kode2 = "W";}
if (value('EI09')==24) {$Kode2 = "X";}
if (value('EI09')==25) {$Kode2 = "Y";}
if (value('EI09')==26) {$Kode2 = "Z";}
}

// PS3 prüfen und ggf. umcodieren;
if (value('EI10')=='-9') {$Kode3 = "--";
} else {
for ($i = 1; $i <= 9; $i++) {
    if (value('EI10')==$i) {$Kode3 = "0".$i;
}}}
if (value('EI10')=='10') {$Kode3 = "10";}
if (value('EI10')=='11') {$Kode3 = "11";}
if (value('EI10')=='12') {$Kode3 = "12";}

// PS4 prüfen und ggf. umcodieren;
if (value('EI11')=='-9') {$Kode4 = "-";
} else {
if (value('EI11')==1) {$Kode4 = "A";}
if (value('EI11')==2) {$Kode4 = "B";}
if (value('EI11')==3) {$Kode4 = "C";}
if (value('EI11')==4) {$Kode4 = "D";}
if (value('EI11')==5) {$Kode4 = "E";}
if (value('EI11')==6) {$Kode4 = "F";}
if (value('EI11')==7) {$Kode4 = "G";}
if (value('EI11')==8) {$Kode4 = "H";}
if (value('EI11')==9) {$Kode4 = "I";}
if (value('EI11')==10) {$Kode4 = "J";}
if (value('EI11')==11) {$Kode4 = "K";}
if (value('EI11')==12) {$Kode4 = "L";}
if (value('EI11')==13) {$Kode4 = "M";}
if (value('EI11')==14) {$Kode4 = "N";}
if (value('EI11')==15) {$Kode4 = "O";}
if (value('EI11')==16) {$Kode4 = "P";}
if (value('EI11')==17) {$Kode4 = "Q";}
if (value('EI11')==18) {$Kode4 = "R";}
```

```
if (value('EI11')==19) {$Kode4 = "S";}
if (value('EI11')==20) {$Kode4 = "T";}
if (value('EI11')==21) {$Kode4 = "U";}
if (value('EI11')==22) {$Kode4 = "V";}
if (value('EI11')==23) {$Kode4 = "W";}
if (value('EI11')==24) {$Kode4 = "X";}
if (value('EI11')==25) {$Kode4 = "Y";}
if (value('EI11')==26) {$Kode4 = "Z";}
}

// PS5 prüfen und ggf. umcodieren;
if (value('EI12')=='-9') {$Kode5 = "-";
} else {
for ($i = 1; $i <= 9; $i++) {
    if (value('EI12')==$i) {$Kode5 = $i;
}}}
if (value('EI12')=='10') {$Kode5 = "0";}

// Pat-ID aus Einzelvariablen zusammenstellen;
put('patcode',$Kode1.$Kode2.$Kode3.$Kode4.$Kode5);
```

Seite 07

kontakt

PHP-Code

```
$ref = strtoupper(reference()); //reference-Wert in Großbuchstaben umwandeln
if (reference() != '') {put('patcode', $ref);} //reference-Wert in patcode übernehmen
```

Seite 08

SY03

Filterfrage! Filterfrage: Wenn bei Frage SY43 Angabe: 5-10 und richtiges Alter (Frage EI50) und Z.n. PCR-bestätigtem COVID (Frage SY03); Link zum Terminvergabetool sofort teilen, sonst Vetröstungsemail schreiben)

Im Folgenden befragen wir Sie nochmal etwas genauer zu Ihrer COVID-Infektion.

Hatten Sie bereits eine Corona Erkrankung?

- ☐ Nein.
- ☐ Ja, durch einen PCR Test bestätigt (Abstrich, PCR Labortest): Datum des positiven Tests
- ☐ Ja, durch einen Antikörper Test bestätigt (Blutabnahme).
- ☐ Ja, durch einen Antigen-Schnelltest bestätigt (Abstrich, Ergebnis innerhalb von Minuten).
- ☐ Ich denke ja, aber es wurde nicht getestet.
- ☐ Sonstiges:

1 aktive(r) Filter

Filter SY03/F1
Wenn eine der folgenden Antwortoption(en) ausgewählt wurde: **2, 3, 4**
Dann Seite(n) **sym, symlc** des Fragebogens anzeigen (sonst ausblenden)

SY04

Wann traten die ersten Corona-Symptome auf bzw. wann haben Sie gemerkt, dass Sie Corona haben?

Bitte tragen Sie das ungefähre Datum ein.

SY05

Wie stark waren Ihre Symptome während der Corona Infektion?

Symptome während der Corona Infektion

Keine Symptome (0)	1	2	3	4	5	6	7	8	9	Stärkste Symptome (10)
○	○	○	○	○	○	○	○	○	○	○

SY06

Wurden Sie im Krankenhaus behandelt?

- ☐ Nein
- ☐ Ja, auf einer Normalstation
- ☐ Ja, auf einer Intensivstation

SY07

Wie war der Verlauf der Erkrankung?

- ○ Ich bin wieder vollständig genesen, ich fühle mich wieder gesund.
- ○ Ich hatte länger als 4 Wochen Symptome und fühle mich jetzt wieder gesund.
- ○ Ich fühle mich nicht wieder gesund, ich habe weiterhin Symptome.

1 aktive(r) Filter

Filter SY07/F1
Wenn eine der folgenden Antwortoption(en) ausgewählt wurde: **1**
Dann nach dem Klick auf "Weiter" direkt zur Seite **impf1** springen

Seite 10

symlc

SY09

Welche Symptome erleben Sie seit der COVID Erkrankung (erstmalig nach Erkrankung aufgetreten)?

(Mehrfachauswahl möglich)

	Keine Symptome (0)										Stärkste Symptome (10)	Weiß ich nicht
Atemnot	○	○	○	○	○	○	○	○	○	○	○	○
Husten	○	○	○	○	○	○	○	○	○	○	○	○
Herzrasen	○	○	○	○	○	○	○	○	○	○	○	○
Engegefühl in der Brust	○	○	○	○	○	○	○	○	○	○	○	○
Schmerzen in der Brust	○	○	○	○	○	○	○	○	○	○	○	○
Fatigue/Erschöpfung	○	○	○	○	○	○	○	○	○	○	○	○
Fieber	○	○	○	○	○	○	○	○	○	○	○	○
Schmerzen	○	○	○	○	○	○	○	○	○	○	○	○
Konzentrationsschwäche, „Brain fog“, Vergesslichkeit	○	○	○	○	○	○	○	○	○	○	○	○
Kopfschmerzen	○	○	○	○	○	○	○	○	○	○	○	○
Schlafstörungen	○	○	○	○	○	○	○	○	○	○	○	○
Gefühlsstörungen an Armen und Beinen (Kribbeln, Nadelstiche, ...)	○	○	○	○	○	○	○	○	○	○	○	○
Schwindel	○	○	○	○	○	○	○	○	○	○	○	○
Verwirrtheit	○	○	○	○	○	○	○	○	○	○	○	○
	Keine Symptome										Stärkste Symptome	Weiß ich nicht

	(0)										(10)	
Bauchschmerzen	○	○	○	○	○	○	○	○	○	○	○	○
	Keine Symptome (0)										Stärkste Symptome (10)	Weiß ich nicht
Übelkeit	○	○	○	○	○	○	○	○	○	○	○	○
	Keine Symptome (0)										Stärkste Symptome (10)	Weiß ich nicht
Durchfall	○	○	○	○	○	○	○	○	○	○	○	○
	Keine Symptome (0)										Stärkste Symptome (10)	Weiß ich nicht
Appetitlosigkeit	○	○	○	○	○	○	○	○	○	○	○	○
	Keine Symptome (0)										Stärkste Symptome (10)	Weiß ich nicht
Gelenkschmerzen	○	○	○	○	○	○	○	○	○	○	○	○
	Keine Symptome (0)										Stärkste Symptome (10)	Weiß ich nicht
Muskelschmerzen	○	○	○	○	○	○	○	○	○	○	○	○
	Keine Symptome (0)										Stärkste Symptome (10)	Weiß ich nicht
Depressive Symptome	○	○	○	○	○	○	○	○	○	○	○	○
	Keine Symptome (0)										Stärkste Symptome (10)	Weiß ich nicht
Angst Symptome	○	○	○	○	○	○	○	○	○	○	○	○
	Keine Symptome (0)										Stärkste Symptome (10)	Weiß ich nicht
Tinnitus	○	○	○	○	○	○	○	○	○	○	○	○
	Keine Symptome (0)										Stärkste Symptome (10)	Weiß ich nicht
Ohrenschmerzen	○	○	○	○	○	○	○	○	○	○	○	○
	Keine Symptome (0)										Stärkste Symptome (10)	Weiß ich nicht
Trockener/Rauer Hals	○	○	○	○	○	○	○	○	○	○	○	○
	Keine Symptome (0)										Stärkste Symptome (10)	Weiß ich nicht
Verlust des Geschmacks-/ Geruchsinns	○	○	○	○	○	○	○	○	○	○	○	○
	Keine Symptome (0)										Stärkste Symptome (10)	Weiß ich nicht
Hautausschläge	○	○	○	○	○	○	○	○	○	○	○	○
	Keine Symptome (0)										Stärkste Symptome (10)	Weiß ich nicht
Sonstige	○	○	○	○	○	○	○	○	○	○	○	○

SY10

Wenn sonstige, welche?

Wann traten die Symptome nach der Corona Erkrankung auf? SY08

Bitte tragen Sie das ungefähre Datum ein.

SY11

Hier haben Sie die Möglichkeit, Ihre Symptome und den Krankheitsverlauf genauer zu schildern, falls Sie Corona haben oder hatten.

Seite 11

impf1

IM01

Sind Sie gegen SARS-CoV-2 geimpft?

- ◯ Ja
- ◯ Nein
- ◯ Unbekannt

1 aktive(r) Filter

Filter IM01/F1
Wenn eine der folgenden Antwortoption(en) ausgewählt wurde: **1**
Dann Seite(n) **impf** des Fragebogens anzeigen (sonst ausblenden)

Seite 12
impf

IM04

Erste Impfung

Datum der ersten Impfung

IM02

Erste Impfung

Impfstoff bei der ersten Impfung:

- ◯ Biontech/Moderna
- ◯ Astra Zeneca
- ◯ Johnson&Johnson
- ◯ Anderer

IM05

Zweite Impfung

Datum der zweiten Impfung

IM03

Impfstoff:

- ◯ Biontech/Moderna
- ◯ Astra Zeneca
- ◯ Johnson&Johnson
- ◯ Anderer

IM06

Dritte Impfung

Datum der dritten Impfung

IM07

Impfstoff:

- ◯ Biontech/Moderna
- ◯ Astra Zeneca
- ◯ Johnson&Johnson
- ◯ Anderer

DM14

Nun folgen einige Fragen zu ihrer Person.

DM01

Was ist ihr höchster Schulabschluss bzw. welche Schulform besuchen Sie?

- ◯ keiner/Volksschulabschluss
- ◯ Hauptschulabschluss (oder besuche Hauptschule)
- ◯ Realschulabschluss/POS (Mittlere Reife) (oder besuche Realschule)
- ◯ Abitur/Fachhochschulreife (oder besuche Gymnasium/ Fachhochschule)

DM02

Leben, lernen, arbeiten, wohnen oder haben Sie ihre medizinische Versorgung in Niedersachsen?

- ◯ Ja
- ◯ Nein

DM03

Welchem Geschlecht fühlen Sie sich zugehörig?

- ◯ Weiblich
- ◯ Männlich
- ◯ Divers

DM04

Wie alt sind Sie?

[ ] Jahre

DM13

Leben Kinder (oder andere Jugendliche) unter 18 Jahren in Ihrem Haushalt?

- ◯ Ja
- ◯ Nein

DM05
raus

Sind Sie zurzeit berufstätig oder gehen Sie zur Schule (das werten wir auch als Vollzeitbeschäftigung)?

- ◯ Ja, regelmäßig vollzeitbeschäftigt
- ◯ Ja, regelmäßig teilzeitbeschäftigt (≥ 15 Stunden/Woche)
- ◯ Ja, geringfügig oder unregelmäßig teilzeitbeschäftigt (< 15 Stunden/Woche)
- ◯ Nein (z.B. in Ausbildung, Rentner:in, Elternzeit...)

DM06
raus

Sind Sie in einem Gesundheitsberuf tätig?

- ◯ Ja
- ◯ Nein

DM07

Sind Sie in der Patient:innenversorgung tätig?

- ◯ Ja
- ◯ Nein

DM08

Sind Sie in Deutschland geboren?

- ◯ Ja
- ◯ Nein, in einem anderen EU Land
- ◯ Nein, in einem Land außerhalb der EU

DM09

Was trifft auf Sie zu? (Mehrfachauswahl möglich)

- ☐ Ich bin deutsche:r Staatsbürger:in
- ☐ Ich bin nach 1949 nach Deutschland zugewandert
- ☐ Ein Elternteil oder beide Eltern ist/sind nach 1949 nach Deutschland zugewandert

DM10

Haben Sie Vorerkrankungen?

- ◯ Nein
- ◯ Ja

DM11

Falls Ja, welche? (Mehrfachauswahl möglich)

- ☐ Bluthochdruck
- ☐ Herzinsuffizienz
- ☐ Koronare Herzkrankheit
- ☐ Vorhofflimmern/-flattern
- ☐ andere Herzrhythmusstörungen
- ☐ Diabetes Typ 1
- ☐ Diabetes Typ 2
- ☐ Asthma Bronchiale
- ☐ COPD
- ☐ Niereninsuffizienz
- ☐ Gicht
- ☐ Gallensteinleiden
- ☐ starkes Übergewicht
- ☐ Morbus Crohn / Colitis Ulcerosa
- ☐ Chronische Hepatitis
- ☐ HIV
- ☐ Schuppenflechte / Psoriasis
- ☐ chronische Wunden
- ☐ Allergien
- ☐ Neurodermitis
- ☐ Schilddrüsenerkrankung
- ☐ Rheuma
- ☐ Polymyalgie / Polymyalgia rheumatica
- ☐ andere Autoimmunerkrankungen
- ☐ chronische Schmerzen
- ☐ Migräne
- ☐ Epilepsie/Anfallsleiden
- ☐ Parkinson
- ☐ Demenz
- ☐ Schizophrenie/Manie
- ☐ Depression
- ☐ Durchblutungsstörung der Beine (pAVK)
- ☐ sonstige Erkrankung: ____

DM12
raus

Falls ja, haben oder hatten Sie eine der folgenden Krebserkrankungen? (Mehrfachauswahl möglich)

- ☐ Nein
- ☐ Lungenkrebs
- ☐ Hautkrebs
- ☐ Brustkrebs
- ☐ Darmkrebs
- ☐ Magenkrebs
- ☐ Prostatakrebs
- ☐ andere/sonstige ____

EQ01

EuroQoL

Bitte tippen Sie auf den folgenden Bildschirmseiten die Aussage an, die Ihre Gesundheit HEUTE am besten beschreibt.

EQ02

Beweglichkeit/Mobilität HEUTE

- ◯ Ich habe keine Probleme herumzugehen
- ◯ Ich habe einige Probleme herumzugehen
- ◯ Ich bin ans Bett gebunden

EQ03

Für sich selbst sorgen HEUTE

- ◯ Ich habe keine Probleme, für mich selbst zu sorgen
- ◯ Ich habe einige Probleme, mich selbst zu waschen oder mich anzuziehen
- ◯ Ich bin nicht in der Lage, mich selbst zu waschen oder anzuziehen

EQ04

Alltägliche Tätigkeiten HEUTE

(z. B. Arbeit, Studium, Hausarbeit, Familien- oder Freizeitaktivitäten)

- ◯ Ich habe keine Probleme, meinen alltäglichen Tätigkeiten nachzugehen
- ◯ Ich habe einige Probleme, meinen alltäglichen Tätigkeiten nachzugehen
- ◯ Ich bin nicht in der Lage, meinen alltäglichen Tätigkeiten nachzugehen

EQ05

Schmerzen/Körperliche Beschwerden HEUTE

- ◯ Ich habe keine Schmerzen oder Beschwerden
- ◯ Ich habe einige Schmerzen oder Beschwerden
- ◯ Ich habe extreme Schmerzen oder Beschwerden

EQ06

Angst/Niedergeschlagenheit HEUTE

- ◯ Ich bin nicht ängstlich oder deprimiert
- ◯ Ich bin mäßig ängstlich oder deprimiert
- ◯ Ich bin extrem ängstlich oder deprimiert

EQ08

Gesundheitszustand

	schlechtester Gesundheitszustand (0)	beste Gesundheits (100
Ihr heutiger Gesundheitszustand		

EQ09

© EuroQol Group. EQ-5D™ is a trade mark of the EuroQol Group

ME01

Die folgenden Fragen beziehen sich auf mögliche Beeinträchtigungen im Alltag durch Erkrankungen und die Pandemiesituation.

Bitte kreuzen Sie bei jeder Frage das entsprechende Kästchen an. Dabei bedeuten:

[0]= keine Beeinträchtigung ... ... [10] = keine Aktivität mehr möglich

Ansonsten kreuzen Sie ein entsprechendes Kästchen dazwischen an.

**Bestehen Beeinträchtigungen bei...**	keine Beeinträchtigung (0)										keine Aktivität mehr möglich (10)
üblichen Aktivitäten des täglichen Lebens (dieser Bereich bezieht sich auf Tätigkeiten wie z.B. Waschen, Ankleiden, Essen, sich im Haus bewegen, etc.)	○	○	○	○	○	○	○	○	○	○	○
familiären und häuslichen Verpflichtungen (dieser Bereich bezieht sich auf Tätigkeiten, die das Zuhause oder die Familie betreffen. Er umfasst Hausarbeit und andere Arbeiten rund um das Haus bzw. die Wohnung, auch Gartenarbeit)	○	○	○	○	○	○	○	○	○	○	○
Erledigungen außerhalb des Hauses (dieser Bereich umfasst z.B. Einkäufe, Amtsgänge, Bankgeschäfte auch unter Benutzung üblicher Verkehrsmittel)	○	○	○	○	○	○	○	○	○	○	○
täglichen Aufgaben und Verpflichtungen (dieser Bereich umfasst alltägliche Aufgaben und Verpflichtungen wie z.B. Arbeit, Schule, Hausarbeit)	○	○	○	○	○	○	○	○	○	○	○
Erholung und Freizeit (dieser Bereich umfasst Hobbys, Freizeitaktivitäten und Sport, Urlaub)	○	○	○	○	○	○	○	○	○	○	○
sozialen Aktivitäten (dieser Bereich bezieht sich auf das Zusammensein mit Freunden und Bekannten, wie z.B. Essen gehen, besondere Anlässe, Theater- oder Kinobesuche, etc.)	○	○	○	○	○	○	○	○	○	○	○
engen persönlichen Beziehungen (dieser Bereich bezieht sich auf Eingehen und Aufrechterhalten enger Freundschaften, Partnerschaften, Ehe)	○	○	○	○	○	○	○	○	○	○	○
Sexualleben (dieser Bereich bezieht sich auf die Häufigkeit und die Qualität des Sexuallebens)	○	○	○	○	○	○	○	○	○	○	○

ME02

**Wie stark sind folgende Belastungen?**	kann Belastungen ertragen										kann Belastungen nicht mehr ertragen
Stress und außergewöhnliche Belastungen (dieser Bereich umfasst z.B. familiäre Auseinandersetzungen und andere Konflikte sowie außergewöhnliche Belastungen im Beruf und am Arbeitsplatz)	○	○	○	○	○	○	○	○	○	○	○
Seelische Belastungen durch Pandemiesituation (z.B. Stimmungsschwankungen, Ärger, Depressionen, Angst o.ä.)	○	○	○	○	○	○	○	○	○	○	○

ME03

IMET 2006 © Deck, R., Borowski, C., Mittag, O., Hüppe, A., Raspe, H.

Letzte Seite

Vielen Dank für Ihre Teilnahme!

Wir möchten uns ganz herzlich für Ihre Mithilfe bedanken.

Ihre Antworten wurden gespeichert, Sie können das Browser-Fenster nun schließen.

info@defeat-corona.de – 2021
