# Supplementary material for: Association of long COVID with health-related Quality of Life and Social Participation in Germany: Finding from an online-based cross-sectional survey
Source: Heliyon. 2024 Feb 9;10(4):e26130. doi: 10.1016/j.heliyon.2024.e26130 (PMC10877341; doi:10.1016/j.heliyon.2024.e26130)

# Supplementary information – Measures

**EQ-5D-3L English Version**


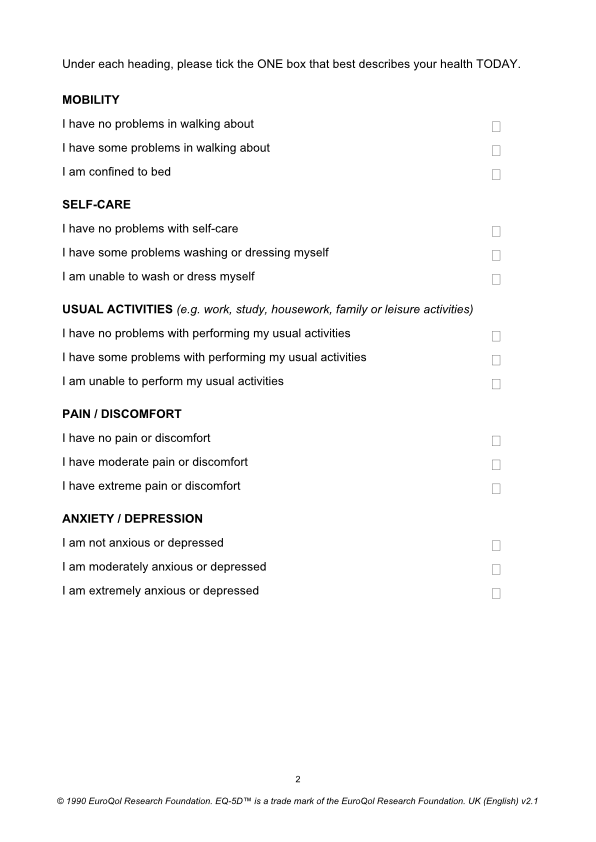

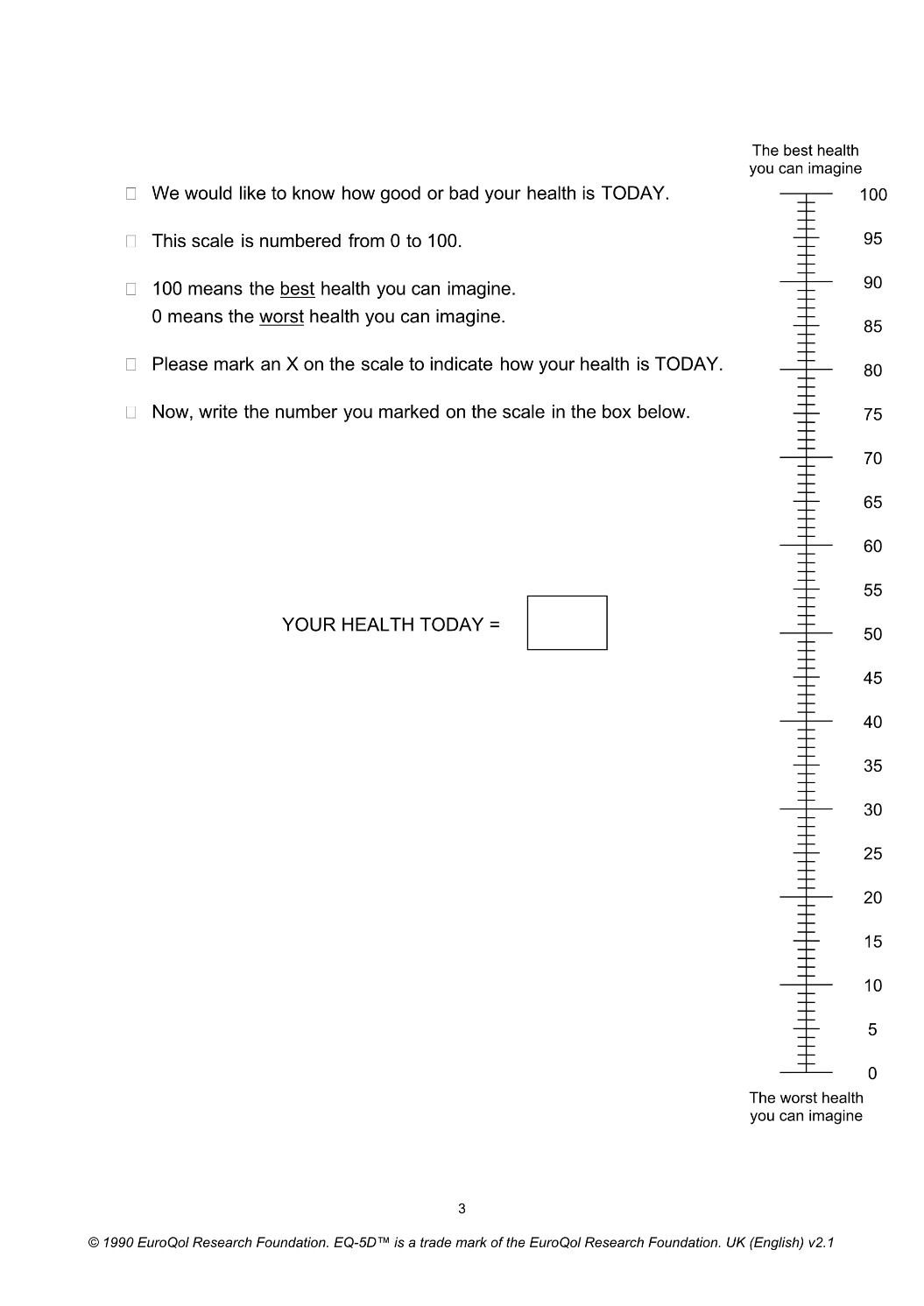


**EQ-5D-3L German translation**


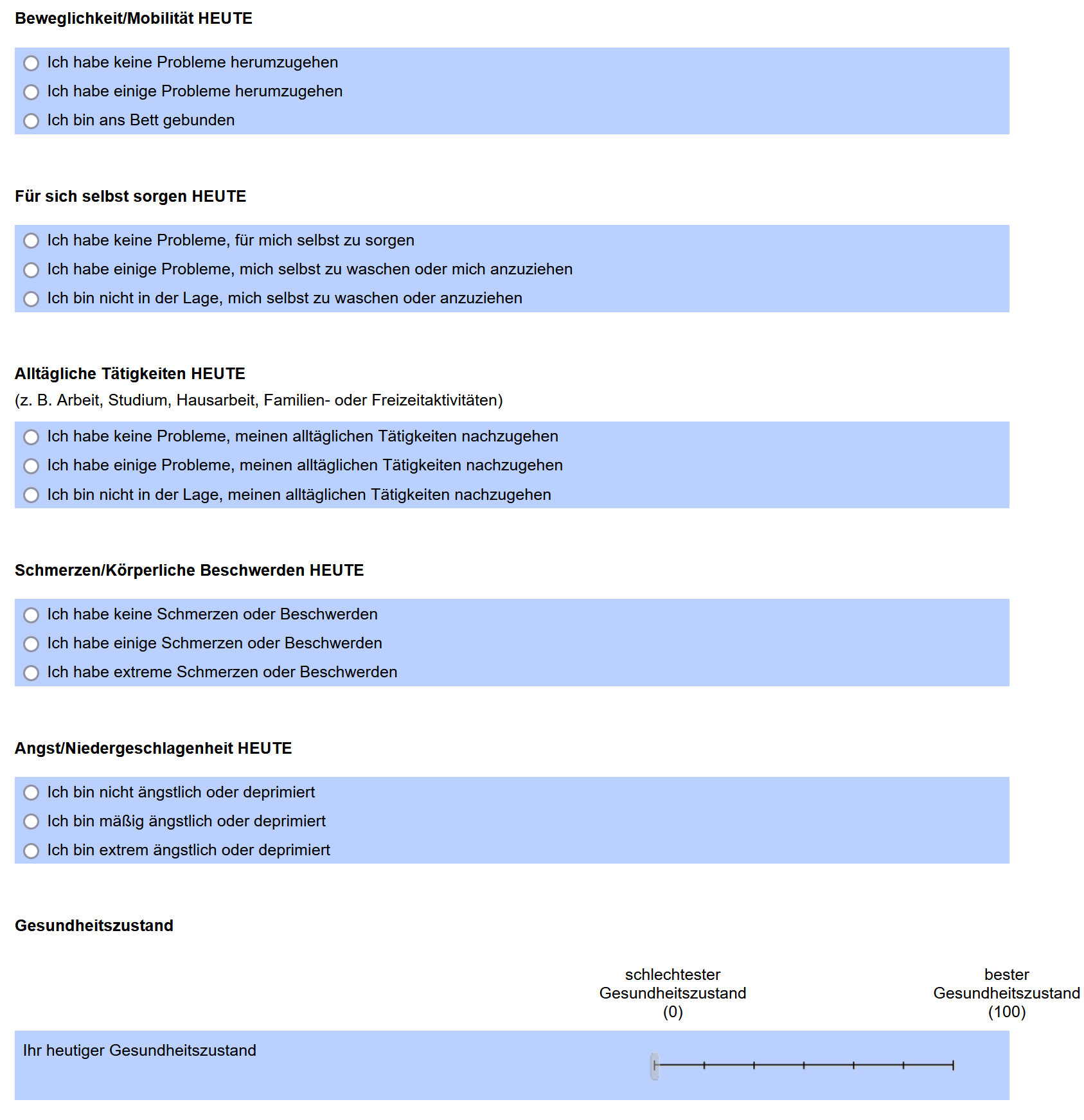


**Index for the Assessment of Health Impairments (English translation (not validated))**


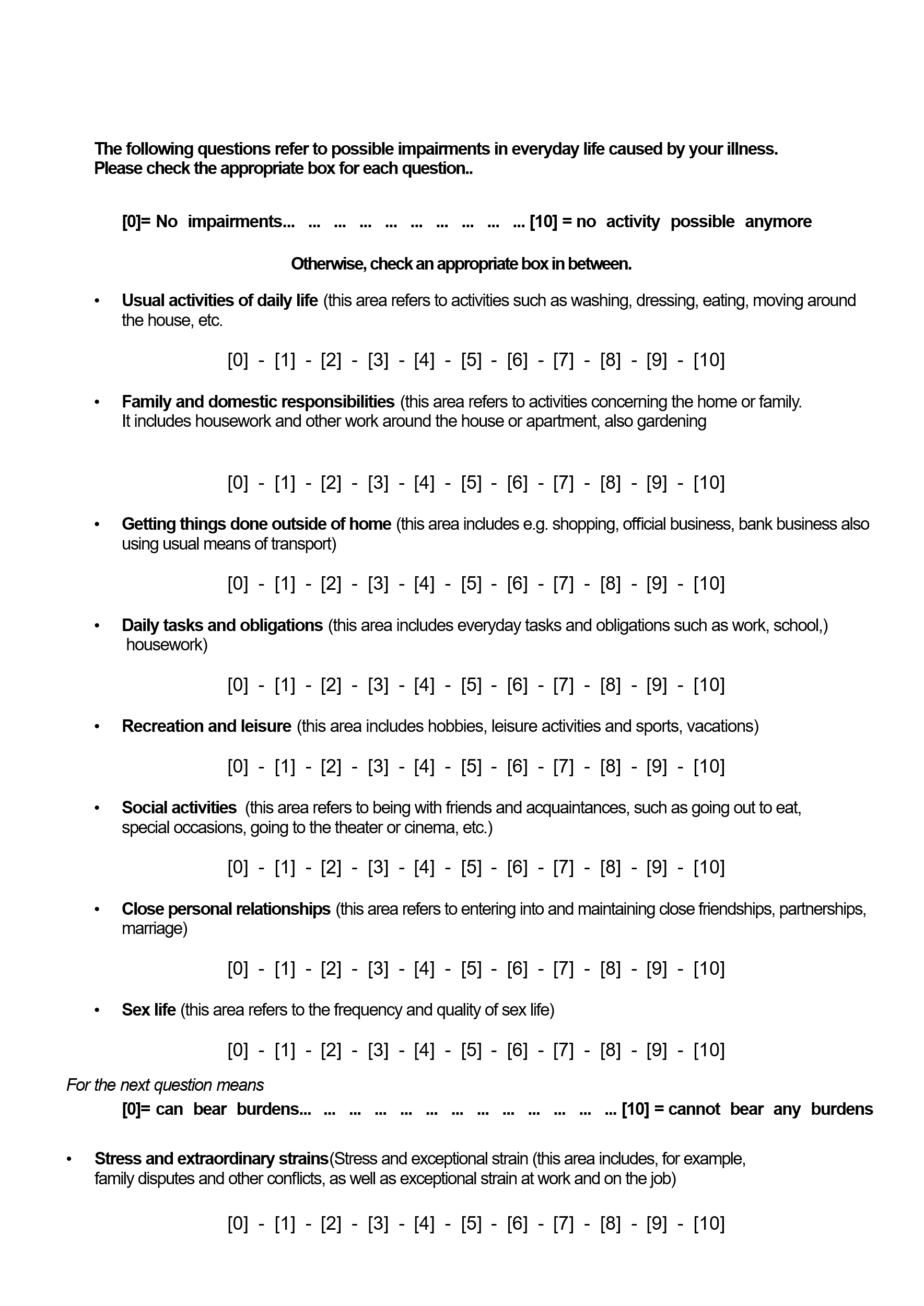


**Index for the Assessment of Health Impairments (German version (validated))**


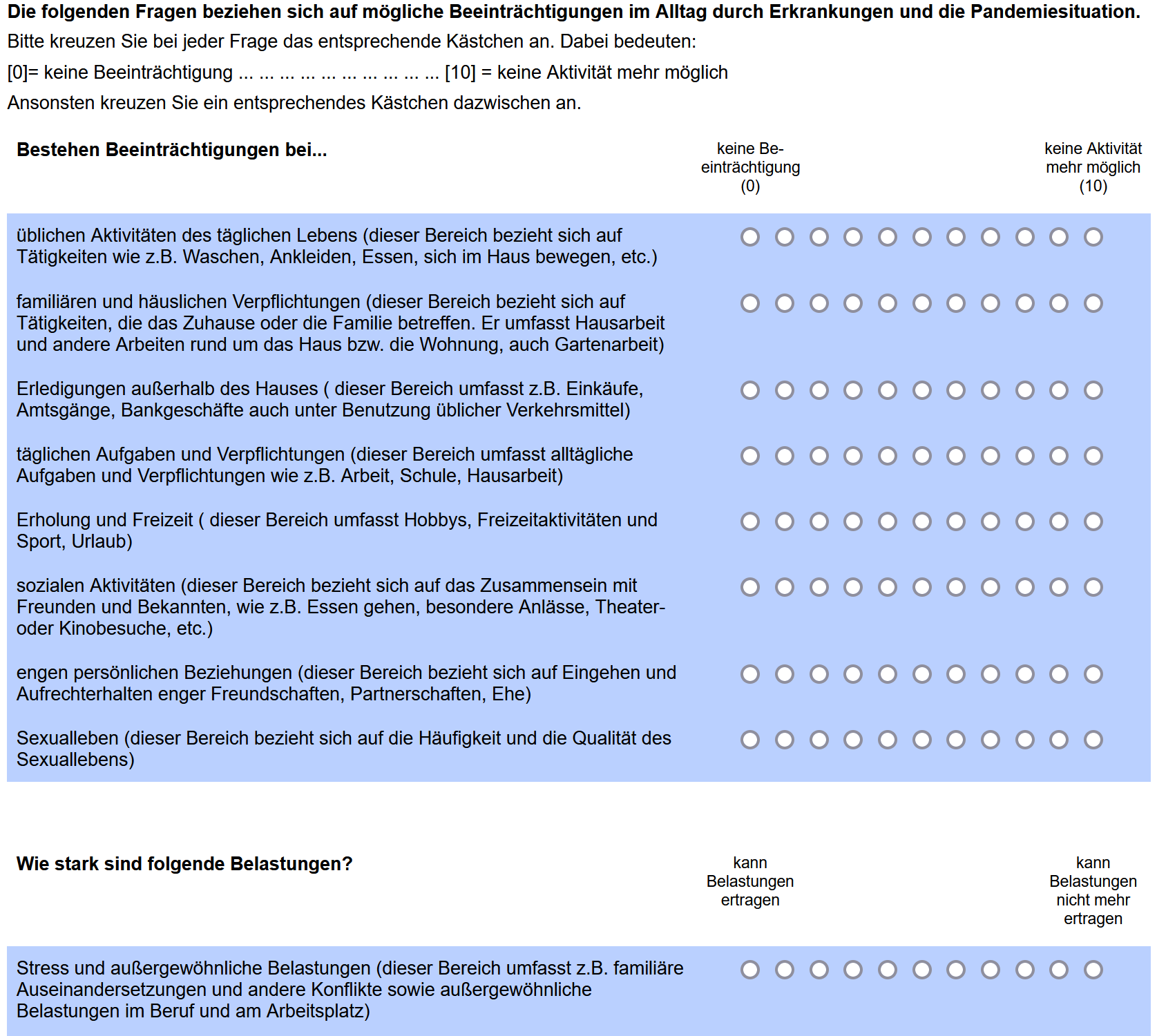

Supplement: Multimedia component 2 [file mmc2.docx]
