# Supplementary material for: Association of long COVID with health-related Quality of Life and Social Participation in Germany: Finding from an online-based cross-sectional survey
Source: Heliyon. 2024 Feb 9;10(4):e26130. doi: 10.1016/j.heliyon.2024.e26130 (PMC10877341; doi:10.1016/j.heliyon.2024.e26130)
Supplement: Multimedia component 3 [file mmc3.docx]

# Supplementary information

Table SI 1: Correlation between EQ-5D-3L index values, EQ-5D VAS scores and IMET Scores

|  | IMET | IMET1 | IMET2 | IMET3 | IMET4 | IMET5 | IMET6 | IMET7 | IMET8 | IMET9 |
| --- | --- | --- | --- | --- | --- | --- | --- | --- | --- | --- |
| Index | -0.71 | -0.58 | -0.72 | -0.63 | -0.70 | -0.57 | -0.50 | -0.49 | -0.55 | -0.56 |
| VAS | -0.69 | -0.58 | -0.73 | -0.64 | -0.69 | -0.58 | -0.46 | -0.45 | -0.52 | -0.51 |

All correlations significant p <0.05; Spearman's rank correlation used

Index: EQ-5D-3L index values; VAS: EQ-5D VAS scores; IMET1: Usual activities of daily life; IMET2: Family and domestic responsibilities; IMET3: Getting thing done outside of home; IMET4: Daily tasks and obligations; IMET5: Recreation and leisure; IMET6: Social activities; IMET7: Close personal relationships; IMET8: Sex life; IMET9: Stress and extraordinary strain

Table SI 2: EQ-5D-3L health profiles by COVID-19 status with corresponding mean EQ-5D VAS score

| All (n=2,875) | Lg COVID (n=1,421) | ExCOVID (n=260) | NoCOVID (n=1,507) |
| --- | --- | --- | --- |
| 11111 (34.9) \| 88.3 | 11222 (16.7) \| 56.7 | 11111 (60.0) \| 90.5 | 11111 (51.1) \| 88.6 |
| 11121 (10.0) \| 78.4 | 21222 (9.9) \| 49.6 | 11112 (15.0) \| 85.0 | 11121 (11.4) \| 81.1 |
| 11222 (8.7) \| 56.8 | 11111 (9.8) \| 82.7 | 11121 (9.6) \| 77.5 | 11112 (10.7) \| 81.5 |
| 11112 (8.2) \| 81.1 | 11221 (9.5) \| 57.2 | 11122 (3.8) \| 81.8 | 11122 (6.6) \| 76.4 |
| 11122 (6.4) \| 73.2 | 11121 (7.8) \| 73.6 | 11222 (2.7) \| 67.1 | 11222 (3.1) \| 58.4 |
| 21222 (5.0) \| 48.3 | 11122 (6.3) \| 68.9 | 11212 (2.3) \| 68.7 | 11221 (1.8) \| 66.9 |
| 11221 (4.7) \| 58.7 | 21221 (4.5) \| 51.8 | 11211 (1.2) \| 85.0 | 21222 (1.7) \| 49.1 |
| 21221 (2.7) \| 51.5 | 11112 (3.3) \| 76.1 | 21221 (0.8) \| 67.5 | 21221 (1.5) \| 49.1 |
| 11212 (1.8) \| 65.6 | 21232 (2.5) \| 37.4 | 11223 (0.8) \| 48.0 | 11212 (1.5) \|72.1 |
| 11223 (1.4) \| 47.7 | 11223 (2.3) \| 48.1 | 21222 (0.8) \| 70.0 | 21122 (1.0) \| 66.2 |

health states according to EQ-5D-3L from left to right (1= no impairments; 2: mild impairments; 3: severe impairments): mobility, self-care, usual activities, pain/discomfort and anxiety/depression (%) | mean EQ5D VAS score

Table SI 3: EQ-5D-3L health Profiles by COVID-19 status with corresponding mean IMET Scores

| All (n=2,875) | Lg COVID (n=1,421) | ExCOVID (n=260) | NoCOVID (n=1,507) |
| --- | --- | --- | --- |
| 11111 (34.9) \| 10.5 | 11222 (16.7) \| 35.9 | 11111 (60.0) \| 8.1 | 11111 (51.1) \| 11.2 |
| 11121 (10.0) \| 13.2 | 21222 (9.9) \| 43.5 | 11112 (15.0) \| 14.8 | 11121 (11.4) \| 13.4 |
| 11222 (8.7) \| 36.2 | 11111 (9.8) \| 9.1 | 11121 (9.6) \| 16.7 | 11112 (10.7) \| 19.1 |
| 11112 (8.2) \| 17.3 | 11221 (9.5) \| 30.1 | 11122 (3.8) \| 15.4 | 11122 (6.6) \| 21.4 |
| 11122 (6.4) \| 20.2 | 11121 (7.8) \| 12.2 | 11222 (2.7) \| 27.1 | 11222 (3.1) \| 39.0 |
| 21222 (5.0) \| 44.2 | 11122 (6.3) \| 19.4 | 11212 (2.3) \| 26.0 | 11221 (1.8) \| 31.1 |
| 11221 (4.7) \| 30.1 | 21221 (4.5) \| 38.7 | 11211 (1.2) \| 17.3 | 21222 (1.7) \| 47.8 |
| 21221 (2.7) \| 40.0 | 11112 (3.3) \| 13.5 | 21221 (0.8) \| 24.5 | 21221 (1.5) \| 45.2 |
| 11212 (1.8) \| 27.7 | 21232 (2.5) \| 52.9 | 11223 (0.8) \| 40.0 | 11212 (1.5) \| 26.4 |
| 11223 (1.4) \| 46.6 | 11223 (2.3) \| 47.6 | 21222 (0.8) \| 42.0 | 21122 (1.0) \| 28.2 |

health states according to EQ-5D-3L from left to right (1= no impairments; 2: mild impairments; 3: severe impairments): mobility, self-care, usual activities, pain/discomfort and anxiety/depression (%) | mean IMET score
